# Genome-Wide Methylation Profiling in the Thalamus of Scrapie Sheep

**DOI:** 10.3389/fvets.2022.824677

**Published:** 2022-02-16

**Authors:** Adelaida Hernaiz, Arianne Sanz, Sara Sentre, Beatriz Ranera, Oscar Lopez-Pérez, Pilar Zaragoza, Juan J. Badiola, Hicham Filali, Rosa Bolea, Janne M. Toivonen, Inmaculada Martín-Burriel

**Affiliations:** ^1^Laboratorio de Genética Bioquímica (LAGENBIO), Facultad de Veterinaria, Universidad de Zaragoza-IA2, IIS, Zaragoza, Spain; ^2^Facultad de Ciencias de la Salud, Universidad San Jorge, Zaragoza, Spain; ^3^Centro de Encefalopatías y Enfermedades Transmisibles Emergentes (CEETE), Facultad de Veterinaria, Universidad de Zaragoza-IA2, IIS, Zaragoza, Spain; ^4^Centro de Investigación Biomédica en Red de Enfermedades Neurodegenerativas (CIBERNED), Instituto de Salud Carlos III, Madrid, Spain

**Keywords:** DNA methylation, thalamus, ovine scrapie, prion, whole genome bisulfite sequencing

## Abstract

Scrapie is a neurodegenerative disorder belonging to the group of transmissible spongiform encephalopathy (TSE). Scrapie occurs in sheep and goats, which are considered good natural animal models of these TSE. Changes in DNA methylation occur in the central nervous system (CNS) of patients suffering from prion-like neurodegenerative diseases, such as Alzheimer's disease. Nevertheless, potential DNA methylation alterations have not yet been investigated in the CNS of any prion disease model or naturally infected cases, neither in humans nor in animals. Genome-wide DNA methylation patterns were studied in the thalamus obtained from sheep naturally infected with scrapie at a clinical stage (*n* = 4) and from controls (*n* = 4) by performing a whole-genome bisulfite sequencing (WGBS) analysis. Ewes carried the scrapie-susceptible ARQ/ARQ *PRNP* genotype and were sacrificed at a similar age (4–6 years). Although the average genomic methylation levels were similar between the control and the scrapie animals, we identified 8,907 significant differentially methylated regions (DMRs) and 39 promoters (DMPs). Gene Ontology analysis revealed that hypomethylated DMRs were enriched in genes involved in transmembrane transport and cell adhesion, whereas hypermethylated DMRs were related to intracellular signal transduction genes. Moreover, genes highly expressed in specific types of CNS cells and those previously described to be differentially expressed in scrapie brains contained DMRs. Finally, a quantitative PCR (qPCR) validation indicated differences in the expression of five genes (*PCDH19, SNCG, WDR45B, PEX1*, and *CABIN1*) that matched the methylation changes observed in the genomic study. Altogether, these results suggest a potential regulatory role of DNA methylation in prion neuropathology.

## Introduction

Prion diseases are fatal and transmissible neurodegenerative disorders that occur in humans and animals (1). These diseases are caused by the conformational conversion of the cellular prion protein (PrP^C^) to an infectious isoform PrP^Sc^, which is partially resistant to proteases and prone to form aggregates (2). The accumulation of PrP^Sc^ in the central nervous system (CNS) causes spongiform neuronal degeneration, activation of glial cells, and neuronal loss (3). Ovine scrapie was the first reported transmissible spongiform encephalopathy (TSE) (4) and has been widely studied. Several transcriptomic studies performed in sheep have reported differentially expressed genes (DEGs) and proteins that seem to be involved in the pathogenesis of scrapie and other neurodegenerative diseases including human prion diseases (5–8). These common findings between scrapie and human prion diseases support the use of scrapie sheep as a good natural animal model to study the molecular mechanisms of prion neuropathology and to identify potential diagnostic and therapeutic biomarkers for prion diseases.

Functional genomics provides important tools to investigate molecular mechanisms of the disease and potential disease biomarkers. In previous studies, using custom arrays, we described significant changes in the CNS transcriptome of sheep naturally infected with scrapie at the early and late stages of the disease (5, 6). Dysregulated genes were associated with ion binding and transport, nucleotide binding, structural molecules, immune system, secreted extracellular proteins, lysosomal proteases, and phospho-proteins.

Gene expression can be modulated by epigenetic mechanisms. DNA methylation at the C5 position of Cytosine (mC) is one of the main epigenetic regulatory mechanisms, which is essential for the adequate development of the organism. Methylation usually occurs in CpG islands (CGIs) located in promoter regions or regulatory domains and also within intergenic regions (9). Environmental variables, such as nutrition and stress exposure, can induce alterations in DNA methylation (10). Many of these may constitute epigenetic drift; that is, they are not translated into phenotypic effect. However, some environmental changes may display a relevant effect in the modulation of gene expression in disease-associated status (11).

Epigenetics regulate neural activity in the brain (12), and DNA methylation seems to be important in memory formation and aging-related cognitive decline (13, 14). Regulation by DNA methylation of specific genes in Alzheimer's disease (AD) (15, 16) and in Parkinson's disease (PD) (17) has been demonstrated. Distinct methylation observed in PD patients involves genes previously associated with the disease, and concordant alterations between the brain and peripheral blood leukocytes have been found (18). Hypermethylation also occurs in the G4C2 repeat expansion in C9orf72, which is the most common known cause of amyotrophic lateral sclerosis (ALS) and frontotemporal lobar degeneration (FTLD). The hypermethylation seems to be associated with the presence of the expansion, which could be responsible for C9orf72 downregulation in the disease (19). Global methylation is also altered in the spinal cord of sporadic ALS patients where both hyper- and hypomethylation can directly modulate the expression of adjacent genes (20).

The abovementioned evidence from other neurodegenerative diseases suggests that DNA methylation status may also contribute to the development of prion diseases. The mouse gene coding PrP^C^ contains a CGI in its promotor that seems to modulate the expression of this protein in a tissue-specific manner (21). However, methylation has not been observed in the promoter of the rat *PRNP* gene, at least in PC12 cells (22). Regarding prion diseases, DNA methylation studies at a genomic level have only been performed in blood from patients with sporadic Creutzfeldt–Jakob disease (sCJD), the most common human prion disease (23). To the best of our knowledge, no genome-wide DNA methylation studies have yet been reported in the CNS of any prion disease models or naturally infected cases. We present here a whole-genome bisulfite sequencing (WGBS) analysis of the thalamus obtained from sheep naturally infected with scrapie. The study revealed a number of differentially methylated regions (DMRs) between the control and scrapie animals, as well as an enrichment of several cellular and molecular functions that could contribute to prion-related neuropathology. We compared these results with previously described transcriptomic changes and performed a gene expression analysis that revealed significant changes in the expression of several genes with differential methylation. Of these, several also correlated with prion-related lesions.

## Materials and Methods

### Animals and Tissue Selection

Thalamus samples from eight Rasa Aragonesa sheep were used for WGBS analysis. Four of them were controls, and the other four were naturally infected with scrapie. All the ewes were aged from 4 to 6 years and carried the ARQ/ARQ genotype for the *PRNP* gene (Supplementary Table S1). These animals correspond to those used previously in an association study between gene expression profiles and scrapie-related lesions in the medulla oblongata of scrapie-infected sheep (5). All scrapie animals displayed clear symptomatology. The time the animals were maintained until sacrifice is shown in Supplementary Table S1. Histopathological lesions related to prion diseases (PrP^Sc^ deposition, neuronal vacuolation, spongiosis, and gliosis) were semiquantified in a previous study (24) and are shown in Supplementary Table S1.

The expression of selected differentially methylated genes (DMGs) was evaluated by quantitative PCR (qPCR) using a different set of thalamus samples, with five tissues obtained from control sheep and eight from naturally infected scrapie animals.

### Whole-Genome Bisulfite Sequencing Library Preparation and Sequencing

Genomic DNA was isolated from the thalamus using the Quick-DNA Midiprep Plus kit (Zymo Research, Irvine, CA, USA). Before library construction, degradation of DNA was checked by agarose gel electrophoresis, DNA purity (260/280 ratio) was assessed using NanoDrop spectrophotometer (Thermo Fisher Scientific, Waltham, MA, USA), and the quality and quantity of DNA were determined using Qubit® 2.0 fluorometer (Life Technologies, Carlsbad, CA, USA).

Sequencing libraries were constructed for the different genomic DNA samples. Approximately 2.5 μg of genomic DNA spiked with 12.5 ng of lambda DNA was fragmented by sonication to 200–400 bp with Biorupter, followed by end repair and adenylation. EZ DNA Methylation-Gold Kit (Zymo Research) was used to treat DNA fragments with bisulfite. Afterwards, fragments were selected by size and PCR amplified using KAPA HiFi HotStart Uracil + ReadyMix (2 × ). Library DNA concentration was firstly quantified with Qubit2.0 and then diluted to 1 ng/?l before checking insert size on Agilent 2100 (Agilent Technologies, Santa Clara, CA, USA) and quantified with more accuracy by qPCR (effective concentration of library >2 nM). Libraries were then sequenced on the Illumina HiSeq Xten platform, and 150-bp paired-end reads were generated according to Illumina's protocol. Raw and processed WGBS data are stored at the National Center for Biotechnology Information (NCBI) GEO Series record GSE184767. Library preparation and WGBS were performed by Novogene (UK) Company Limited (Cambridge, UK).

### Data Analysis

Raw reads were saved as fastq format files, and Trimmomatic (v0.36) tool was used to filter out the contaminated adapter sequence and low-quality reads with default parameters. FastQC (v0.11.3) was also performed on the clean data obtained from trimming. Bisulfite-treated reads were aligned with the reference genome (Oar_v4.0) using the Bismark (v0.12.5) software (25) with default parameters. After alignment, Bismark was used to calculate read coverage and sequencing depth, distribution of genome coverage, distribution of chromosome depth and coverage, and coverage depth of each cytosine site context (CpG, CHH, and CHG, where H = A, C, or G) and to identify methylated sites by comparison of read base and the reference genome base at the same position. The bigWig format files containing mapping results, as well as corresponding reference genome and gene annotation files, were visualized using the IGV (Integrative Genomic Viewer) software. In order to find accurate methylated sites, sequencing depth ≥5 and q-values ≤ 0.05 were set as thresholds in the analysis (26, 27). For methylated sites, the methylation level was calculated using the formula ML = mCc/(mCc + umCc), where ML represents the methylation level, and mCc and umCc represent the methylated and unmethylated read counts, respectively. The average methylation level of the whole genome was calculated in each sample with 10 kb as a bin. The average methylation level of all the covered cytosine sites on each chromosome was also calculated. The average methylated level in different cytosine contexts was evaluated in different functional genomic regions such as promotor [2-kb region above a transcriptional start site (TSS)], 5′UTR, exon, intron, CGI, and CGI shore. Functional areas of each gene were divided into 20 bins. Finally, motif characteristics around the positions of methylated cytosines were determined in high-methylated (methylation level higher than 75% in CG context and higher than 25% in non-CG context) and low-methylated sites and in all mC sites.

### Differentially Methylated Region Analysis

DMRs between scrapie and control groups were determined by employing the Bsseq (v0.6.2) software from the Bioconductor (v2.13) package (28) (http://www.bioconductor.org/packages/release/bioc/html/bsseq.html) with default parameters. Bsseq is based on the BSmooth algorithm and targeted for WGBS data. As more variations may occur between scrapie samples than between control samples, the variance was estimated using data from the control group. To identify differentially methylated sites, *t*-test and quantile-based screening (cutoff set as [2.5%, 97.5%], namely, false discovery rate (FDR) < 0.05) were performed. Differentially methylated sites were merged and filtered to obtain the final DMRs with a threshold of at least 0.1 difference in methylation level and at least 3 cytosine sites in every DMR, being two adjacent cytosine sites not beyond 300 bp. DMRs were annotated when overlapping with functional elements (exon, intron, or promoters) of associated genes.

### Differentially Methylated Promoter Analysis

Differentially methylated promoters (DMPs) were identified by testing for each cytosine in each context (CG, CHG, and CHH) of the promoter region using Fisher's exact test. Then, the p-value was corrected applying an FDR < 0.05 and an absolute difference of the methylation levels between the scrapie and the control group >0.2. The promoter region was set as the upstream 2 kb of TSS. Hierarchical clustering methods were adopted to analyze the methylation level of DMPs in scrapie and control thalami.

### Enrichment Analysis

Gene Ontology (GO; http://www.geneontology.org/) and Kyoto Encyclopedia of Genes and Genomes (KEGG) pathways analysis were performed to identify the significant function and pathways of genes associated with DMRs and DMPs (DMGs). GO and KEGG terms with corrected *p* < 0.05 were considered significantly enriched by DMGs.

To investigate if DMGs were expressed in a specific type of CNS cell, a comparison of DMGs with proteins defined as tenfold more abundant in oligodendrocytes, astrocytes, microglia, and cortical neurons in the mouse brain (29) was performed using the InteractiVenn software (30). Afterwards, an enrichment analysis in Reactome (31) was conducted in order to reveal possible pathways with DMGs.

### New Microarray Proof Annotation and Comparison With Identified Differentially Methylated Regions

In a previous study using custom arrays, significant changes in the expression profile of several genes were found in the CNS of sheep naturally infected with scrapie compared to healthy animals (5). As the tissues of this former report belonged to the same animals used in our study, the datasets that contained differentially expressed probes between symptomatic and non-symptomatic animals were used to perform a new annotation. The BLAST-Like Alignment Tool (BLAT) (32) was used to identify the chromosome, the strand, and the location of the beginning and the end of the alignment from the probe in the reference Oar v3.1 genome. That information was included as new information in the original dataset.

Subsequently, the data were loaded into a data.frame object in R (version 3.6.1), and the biomaRt (33, 34) package was used to retrieve annotation information and identifier cross-references from Ensembl. Oar v3.1 reference genome from Ensembl was used as the dataset. Attributes to query the database were as follows: chromosome_name, start_position, end_position, strand, external_gene_name, refseq_mrna_predicted, refseq_mrna, description, external_synonym, and wikigene_name in order to obtain the annotated gene for the probe. Subsequently, annotated genes were included in the new dataset and exported into a comma-separated file.

Those probe sequences without any hit by biomaRt were manually annotated, using the Genome Data Viewer from the NCBI and, again, the Oar v3.1 reference genome (https://www.ncbi.nlm.nih.gov/genome/gdv/browser/genome/?id=GCF_000298735.1).

These genes differentially expressed in scrapie animals were then compared with the identified DMRs using the InteractiVenn software (30).

### RNA/cDNA Preparation and Quantitative Real-Time PCR Analysis

Real-time qPCR was used to validate the biological functionality of a set of DMRs and DMPs by analyzing the expression levels of their linked DMGs. DMRs were selected for validation according to their level of methylation variation, the functionality of their associated genes, and their position in regulatory regions. Similarly, we chose DMPs taking into account the role of DMGs, the number of differentially regulated cytosines, and the level of change.

Total RNA was isolated using the RNeasy Lipid Tissue Mini kit (Qiagen, Valencia, CA, USA). The quality and quantity of RNA were determined using a NanoDrop instrument (Thermo Fisher Scientific, Waltham, MA, USA). cDNA samples were synthesized from 200 ng of total RNA using the reverse transcription (RT) reagent qScript cDNA SuperMix (Quantabio, Beverly, MA, USA). All procedures were performed following the manufacturer's recommended instructions.

The primers used for qPCR are listed in Supplementary Table S2. The reactions were performed on a QuantStudio 3 Real-Time PCR System (Thermo Fisher Scientific). Each PCR was performed by triplicate in a total volume of 10 μl, using 2 μl of cDNA, 300 nM of each primer, and Fast SYBR™ Green Master Mix (Applied Biosystems, Thermo Fisher Scientific). The comparative quantification of the results was standardized by the 2^−Δ*ΔCt*^ method (35), using the geometric mean of *GAPDH, G6PDH*, and *SDHA* as a normalizer (36). Student's *t*-test was applied to identify differences between groups, which were considered significant at *p* < 0.05.

### Correlation Between Methylation Levels, Gene Expression, and Prion-Related Lesions

Tissues analyzed in this work were used in previous studies to validate expression changes of candidate genes in different CNS areas, including the thalamus (24, 37). In these previous works, PrP^Sc^ deposition profiles, neuronal vacuolization, neuropil spongiosis, and gliosis were evaluated and semiquantitatively scored. These published scores were used to analyze any possible relationship between the degree of lesions and whole methylation levels in the different mC context or expression levels of DMGs using Pearson's correlation.

## Results

### DNA Methylation Patterns

Between 832,778,776 and 1,152,563,414 sequencing raw reads were obtained per sample corresponding to 125 and 173 Gb of raw data, respectively. Supplementary Table S3 shows the data quality of the resulting sequences. More than 97.5% of bases displayed a quality higher than Q20, and the percentage of bases showing higher quality (Q30) was higher than 93.7%. The percentage of bisulfite conversion was higher than 99.88% in all samples analyzed.

After raw data cleaning and trimming, the reads were mapped to the reference genome (Oar_v4.0). Between 258,079,070 and 339,064,958 clean reads were mapped on the genome with a percentage of unique mapping reads higher than 53% and covering more than 25 times the genome (Table 1). The percentage of genome bases with a minimum 5 × coverage was higher than 85% and higher than 73% for a 10 × coverage.

**Table 1:** Overview of mapping and genome coverage.

**Sample**	**Mapped reads**	**Unique mapping rate (%)^**a**^**	**Duplication rate (%)^**b**^**	**Sites coverage mean^**c**^**	**5 × coverage^**d**^**	**10 × coverage^**e**^**
C1	339,064,958	59.64	15.94	31.94	85.26	74.66
C2	280,226,053	53.03	13.26	27.23	85.04	73.82
C3	298,189,223	69.19	13.11	29.07	86.39	75.28
C4	287,338,062	55.24	12.21	28.30	85.30	74.55
Sc1	294,158,670	71.52	11.76	29.15	86.79	76.62
Sc2	289,367,126	64.95	11.7	28.67	85.02	74.01
Sc3	292,893,236	71.15	12.13	28.84	86.22	75.75
Sc4	258,079,070	61.95	13.51	24.99	86.10	74.13

a *Percentage of uniquely mapped reads in the filtered clean reads used for mapping*.

b *Percentage of duplication reads*.

c *Sites coverage mean: the average base coverage of the genome*.

d *Percentage of bases with a minimum 5 × coverage of the genome*.

e *Percentage of bases with a minimum 10 × coverage of the genome*.

**Figure 1:**
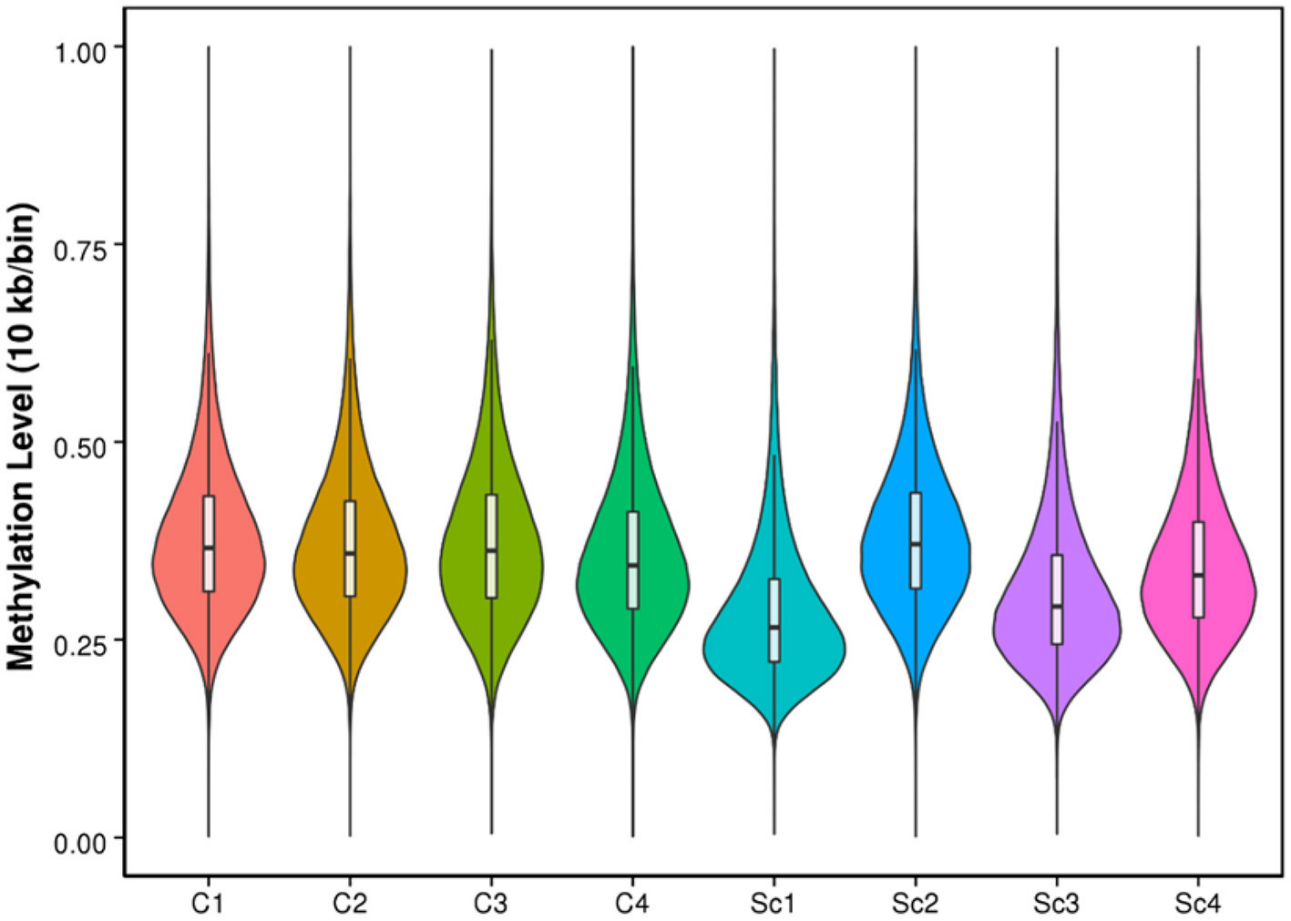
Violin plot for the overall distribution of methylation levels using 10 kb/bin. The abscissa represents the different control (C) and scrapie (S) samples; the ordinate represents the level of methylation of the samples; and the width of each violin represents the density of the point at that methylation level.

No significant differences were observed between scrapie and control sheep in the overall methylation level (Supplementary Table S4) for each cytosine context (mC in CG, CHG, and CHH), nor in the total percentage of methylated cytosines or in the percentages of methylated cytosines in these contexts (Supplementary Table S5), being the higher percentage of mC found in CpG context. Compared with the controls, the scrapie samples displayed higher variability in the percentages of methylated cytosines (Supplementary Table S5) and in the whole-genome methylation level (Figure 1). Sequence preferences flanking the 9 bp sequences around methylated C sites were similar in all samples (Supplementary Figure S1). No specific sequences were found in CG contexts, and CAG and CAC were the most frequent motifs in high and low methylation regions in CHG and CHH contexts, respectively.

### Correlation Between PrP^Sc^ Accumulation and Methylation Levels

**Figure 2:**
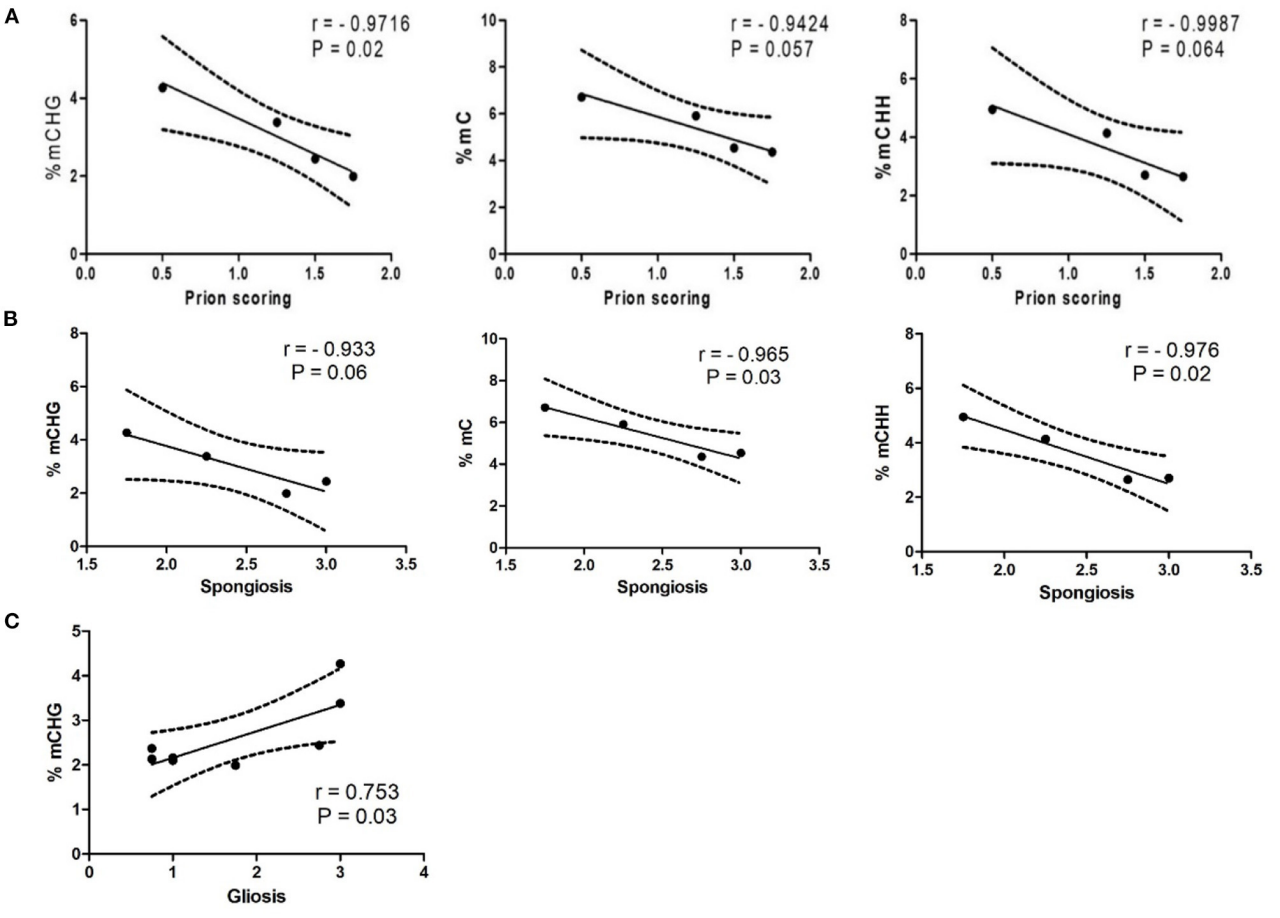
**(A)** Correlation between PrP^Sc^ deposition scores and percentage of methylated cytosines in different motifs. **(B)** Correlation between spongiosis and percentage of methylated cytosines in different motifs. **(C)** Correlation between gliosis and percentage of methylated CHG sites.

In order to explain the variability observed within the scrapie group, we compared global methylated cytosines in the different sites with the degree of PrP^Sc^ deposition in the thalamus within the analyzed animals. We obtained a significant negative correlation between PrP^Sc^ deposits and the percentage of methylated CHG sites (*r* = −0.972, *p* = 0.028) and a negative trend correlation with the percentages of mC sites (*r* = −0.942, *p* = 0.057) and mCHH sites (*r* = −0.936, *p* = 0.064) (Figure 2A).

Similarly, significant negative correlations were found between % mC (*r* = −0.965, *p* = 0.03) and % mCHH (*r* = −0.976, *p* = 0.02) and spongiosis in scrapie thalamus and a trend to signification between this lesion and % mCHG (*r* = −0.933, *p* = 0.06) (Figure 2B). On the contrary, a positive correlation was found between % mCHG (*r* = 0.753, *p* = 0.03) and reactive gliosis in the whole set of animals, but not when the group of scrapie sheep were analyzed separately (Figure 2C).

Finally, the time the animal was showing clinical symptoms could also explain the observed variation because animals showing the highest PrP^Sc^ deposition scores are those maintained for longer periods (Supplementary Table S1).

### Differentially Methylated Cytosines, Regions, and Promoters in Scrapie

DNA methylation level was investigated in different genome components including promoters, exons, and introns. Average methylation levels were similar in all samples, with exons having the features with a lower degree of methylation in all samples (Supplementary Figure S2).

**Figure 3:**
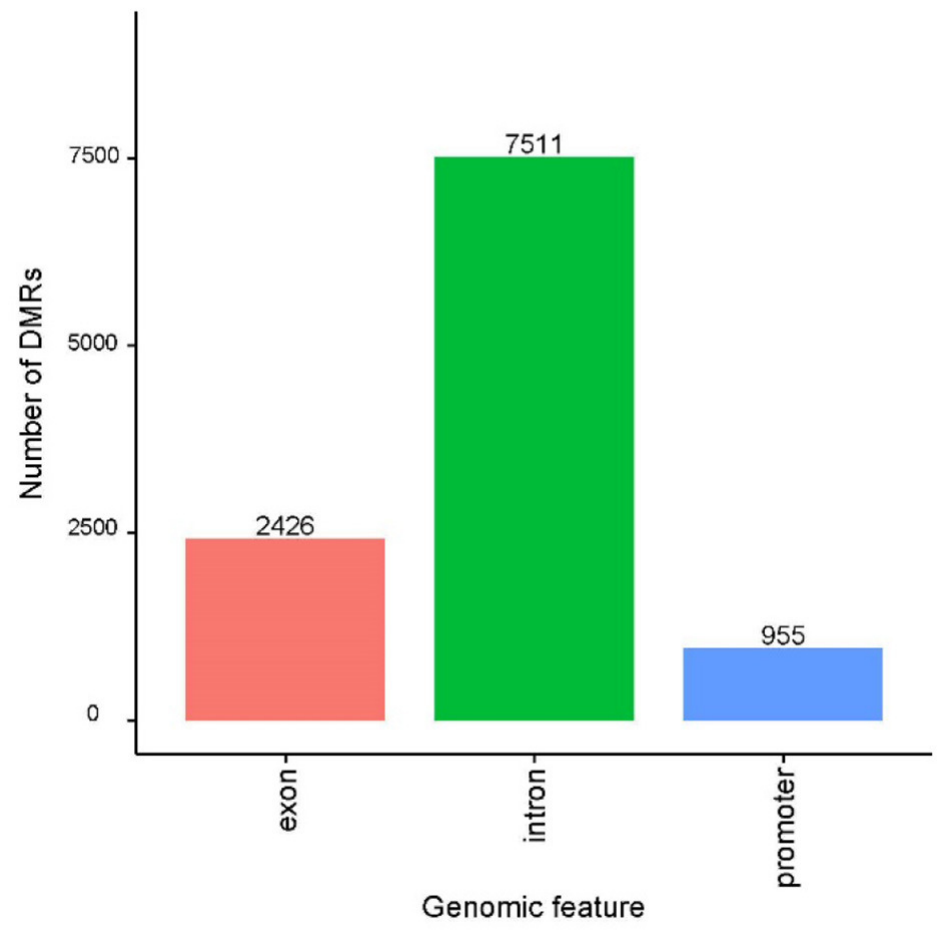
Distribution of differentially methylated regions (DMRs) within functional regions.

Besides this lack of overall methylation changes, DMRs between the control and scrapie groups were identified using the Bsseq package. We identified 8,907 DMRs between scrapie and control tissues, from which 4,630 were hypermethylated and 4,277 hypomethylated (Supplementary Table S6). These DMRs were mainly distributed in introns (7,511), followed by exons (2,426) and 955 located in annotated promoters (Figure 3). A total of 3,568 annotated genes were associated with these DMRs.

As methylation plays an important role in the regulation of gene expression, we performed an analysis to determine DMPs. After filtering and identifying promoters with an absolute difference of the methylation levels >0.2 and with an FDR lower than 0.05, we identified 39 DMPs, 15 of which were hypermethylated and 24 hypomethylated (Supplementary Table S7).

### Enrichment Analysis of Genes Related to Hyper- and Hypomethylated Differentially Methylated Regions

**Figure 4:**
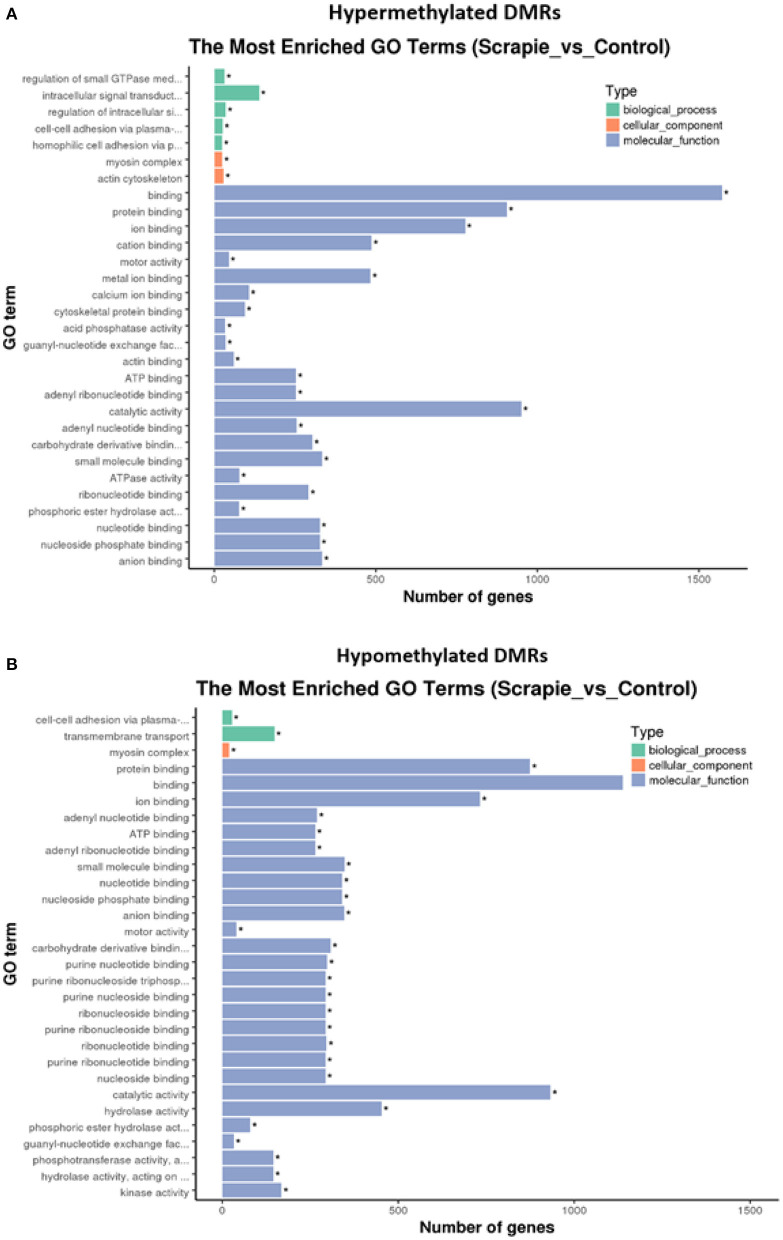
Biological processes, cellular components, and molecular functions enriched in hypermethylated **(A)** and hypomethylated **(B)** differentially methylated regions (DMRs).

In order to determine if different molecular functions can be activated or repressed in scrapie brains, the identified DMRs were directionally separated, and GO enrichment was performed in hypermethylated (Figure 4A, Supplementary Table S8) and hypomethylated (Figure 4B, Supplementary Table S9) DMR-associated genes. Biological processes showing a significant increase of hypermethylated DMRs were related to the regulation of small GTPase mediated signal transduction, intracellular signal transduction and its regulation, homophilic cell adhesion via plasma membrane adhesion molecules, and cell–cell adhesion via plasma–membrane adhesion molecules, the last being also enriched in hypomethylated DMRs. In addition, the transmembrane transport biological process was also enriched in hypomethylated DMRs. Genes related to hyper- and hypomethylated DMRs were significantly enriched for myosin complex cellular components, and genes with hypermethylated DMRs were enriched in components of the actin cytoskeleton. Some molecular functions were found to be enriched in both hyper- and hypomethylated DMRs, such as those related to protein, ATP, ion or ribonucleotide binding, and motor activity. However, hypermethylated DMRs were enriched in calcium ion binding, cytoskeletal protein binding, or acting binding, whereas molecular functions related to purine binding, hydrolase, and kinase activity were enriched in hypomethylated DMRs (Figure 4).

**Figure 5:**
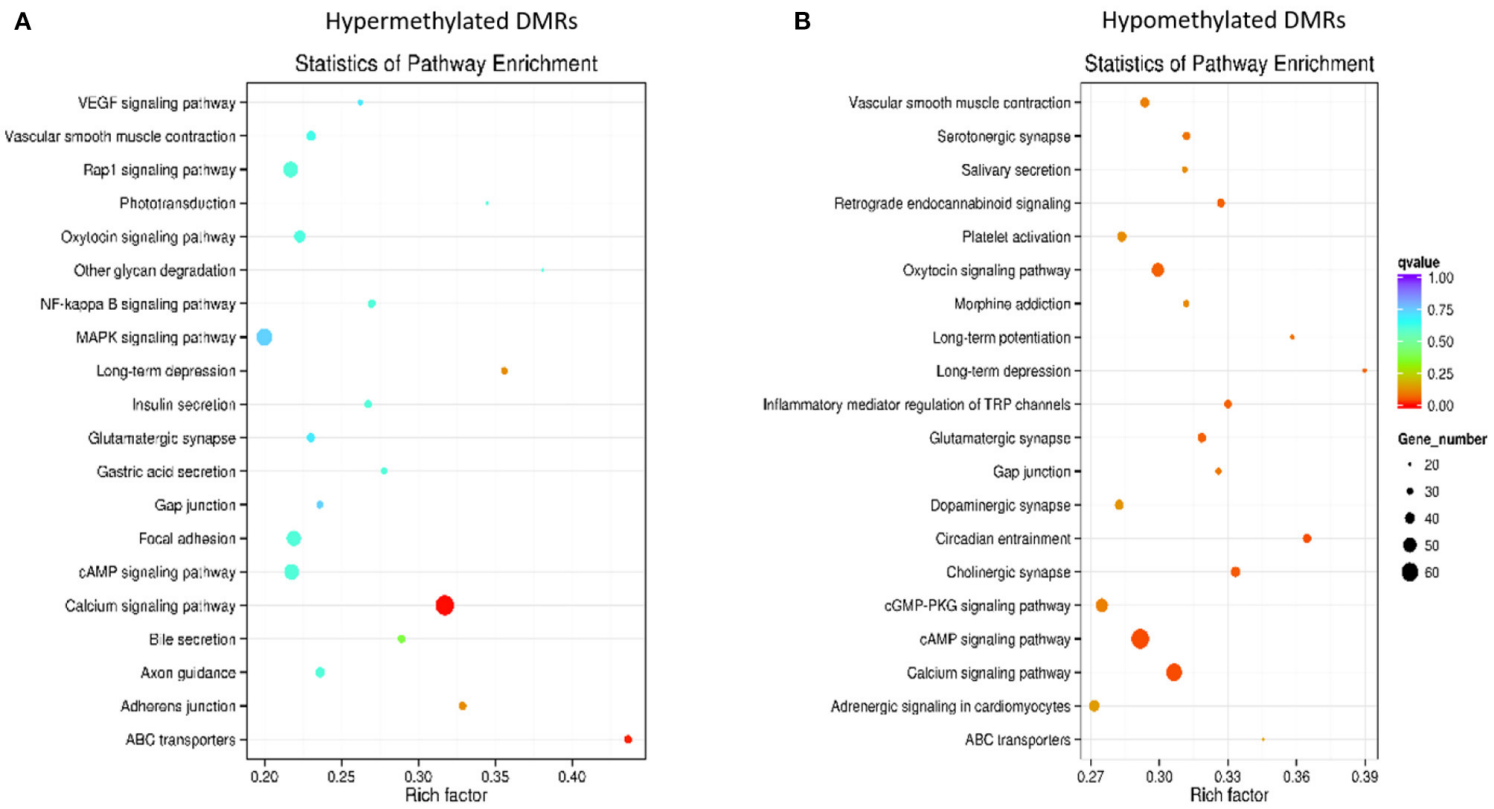
Kyoto Encyclopedia of Genes and Genomes (KEGG) pathway enrichment in hypermethylated **(A)** and hypomethylated **(B)** differentially methylated regions (DMRs).

KEGG pathway analysis revealed a significant enrichment of hypermethylated DMRs in the calcium signaling pathway and ABC transporters (Figure 5A, Supplementary Table S10), whereas hypomethylated DMRs appeared enriched in calcium signaling, circadian entrainment, and cAMP signaling pathways (Figure 5B, Supplementary Table S11).

Due to the relatively low number of DMPs found, GO enrichment analysis did not display any biological process, molecular component, or molecular function with a statistically significant corrected p-value. Although KEGG pathway analysis detected no significant pathways enriched, in hypomethylated DMPs, some pathways displayed a trend toward significance after multiple corrections including terms like apoptosis, lysosomes, protein processing in the endoplasmic reticulum, or AD (Supplementary Table S12).

To investigate if DMGs were expressed in a specific type of CNS cell, we compared DMGs with proteins defined as tenfold more abundant in oligodendrocytes, astrocytes, microglia, and cortical neurons in the mouse brain (29). A total of 206 DMGs corresponded to genes encoding for abundant proteins in neurons (46% of described abundant proteins). An enrichment analysis in Reactome (31) revealed enriched pathways corresponding to unblocking of NMDA receptors, glutamate binding, and activation (Supplementary Table S13). In the analysis of abundant proteins in microglia, we found a total of 83 DMGs (32% of abundant proteins in these cells). Three Reactome pathways were enriched in this set of DMGs: immune system, innate immune system, and neutrophil degranulation (Supplementary Table S13). More than half of the genes encoding proteins abundant in oligodendrocytes displayed DMRs, although we did not find any enriched pathways. Finally, close to 40% of genes encoding known abundant proteins in astrocytes had DMRs. Pathways enriched were related to laminin interactions, post-translational protein phosphorylation, IL6 signaling, and extracellular matrix organization and interactions (Supplementary Table S13).

### Microarray-Identified Genes Differentially Expressed in Scrapie Contain Differentially Methylated Regions

**Figure 6:**
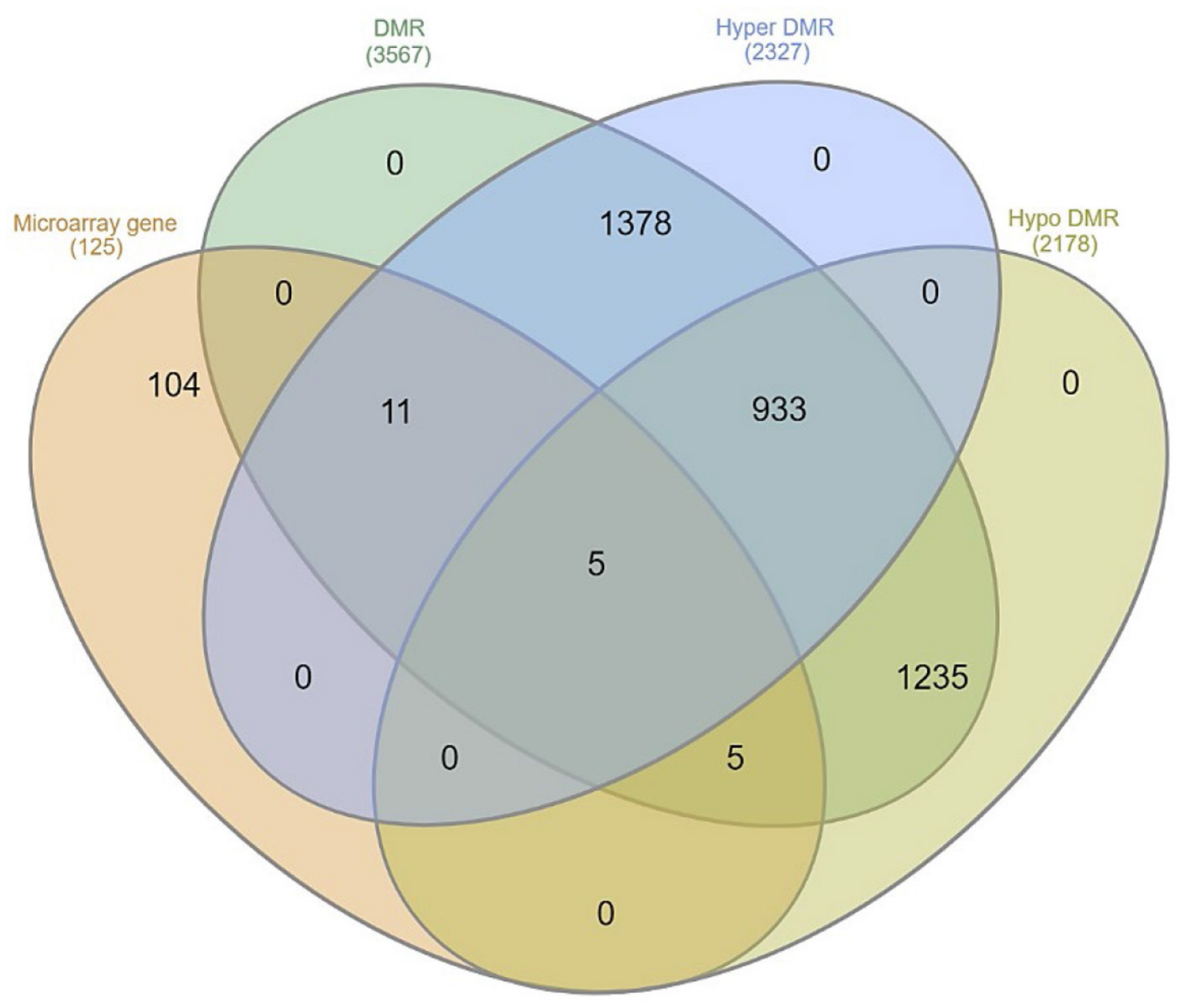
Microarray-annotated genes containing differentially methylated regions (DMRs).

**Figure 7:**
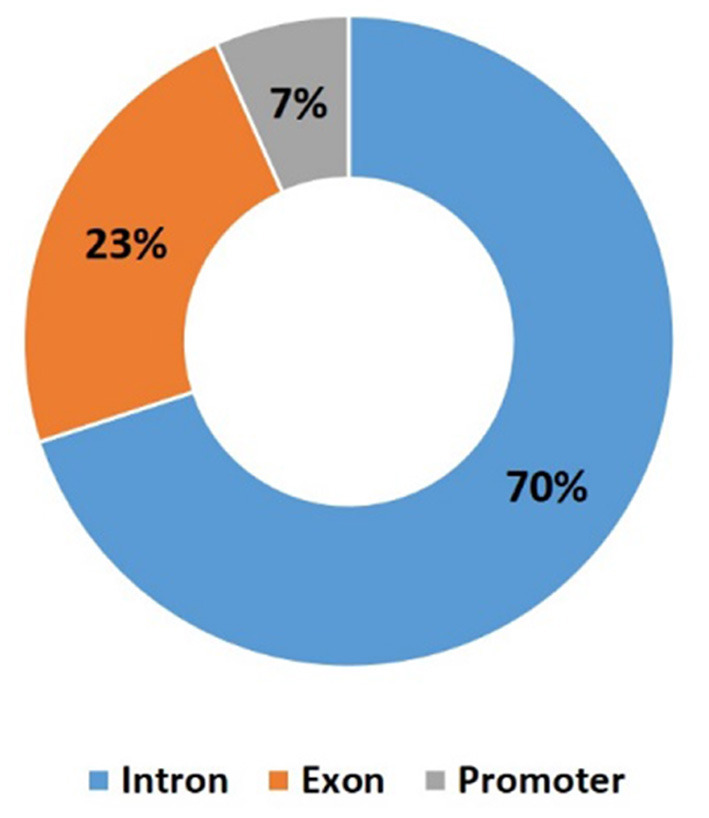
Differentially methylated region (DMR) positions (intron, exon, and promoter) of the microarray-annotated genes.

To identify DEGs previously described in scrapie animals (5) containing DMRs, we enriched the annotation of the published set of DEGs with the identified DMRs. Of the total of annotated genes (*n* = 125) (Supplementary Table S14), 21 were found to harbor DMRs (Figure 6). These DMRs were hyper- or hypomethylated, and the majority of them were located in intron regions (Figure 7).

### Expression Analysis of Genes With Significant Hypo- and Hypermethylated Differentially Methylated Regions and Differentially Methylated Promoters

In order to evaluate the effect of DNA methylation on gene expression in scrapie, we selected, among all the significant hypo- and hypermethylated DMRs and DMPs, a series of genes (DMGs) (Tables 2, 3) with important functions in the nervous system and in other neurodegenerative diseases. The expression of these genes was analyzed by qPCR and correlated with their methylation state and the position (promoter, exon, or intron) of their significant DMRs.

**Table 2:** Expression levels of genes with differentially methylated regions (DMRs).

**Gene**	**DMR methylation state**	**DMR position**	**2^**−ΔΔCt**^**	**Gene expression *p*-value**
A1BG	1 H	Promoter	0.7331	NS
CABIN1	10 H/12 h	Intron/exon	0.7180	*p =* 0.03
CD81	1 H	Promoter	0.7706	NS
MAD1L1	5 H	Promoter/intron	0.8738	NS
Metazoa_SRP	1 h	Exon/promoter	1.1784	NS
PCDH19	1 H	Promoter	0.7022	*p =* 0.03
PEX1	1 H/2 h	Exon	1.3468	*p =* 0.01
PLA2G5	1 H/2 h	Exon	0.9899	NS
PSMG4	2 H	Intron	0.9229	NS
SNX33	2 H	Promoter/intron	0.8682	NS
WDR45B	1 H	Exon	1.1992	*p =* 0.11

*Methylation state, number of DMRs and state (H, hypermethylated, h, hypomethylated); DMR position, positions of the DMR; 2^−ΔΔCt^; relative gene expression in terms of 2^−ΔΔCt^ and gene expression p-value of significant differentially expressed genes; NS, no significant changes*.

**Table 3:** Expression levels of genes with differentially methylated promoters (DMPs).

**Gene**	**DMP methylation** **state**	**2^**−ΔΔCt**^**	**Gene expression** **p-value**
CAPN1	h	1.1886	NS
GSTA4	h	0.8821	NS
INTS1	H	0.9869	NS
KCNK4	h	0.7392	NS
MTSS1L	H	0.8599	NS
NARS	h	0.8130	NS
PLCL2	h	1.2551	NS
SNCG	H	0.3663	*p =* 0.02
WSCD2	H	0.7505	NS

*Methylation state, DMP state (H, hypermethylated, h, hypomethylated); 2^−ΔΔCt^, relative gene expression in terms of 2^−ΔΔCt^ and gene expression p-value of significant differentially expressed genes; NS, no significant changes*.

**Figure 8:**
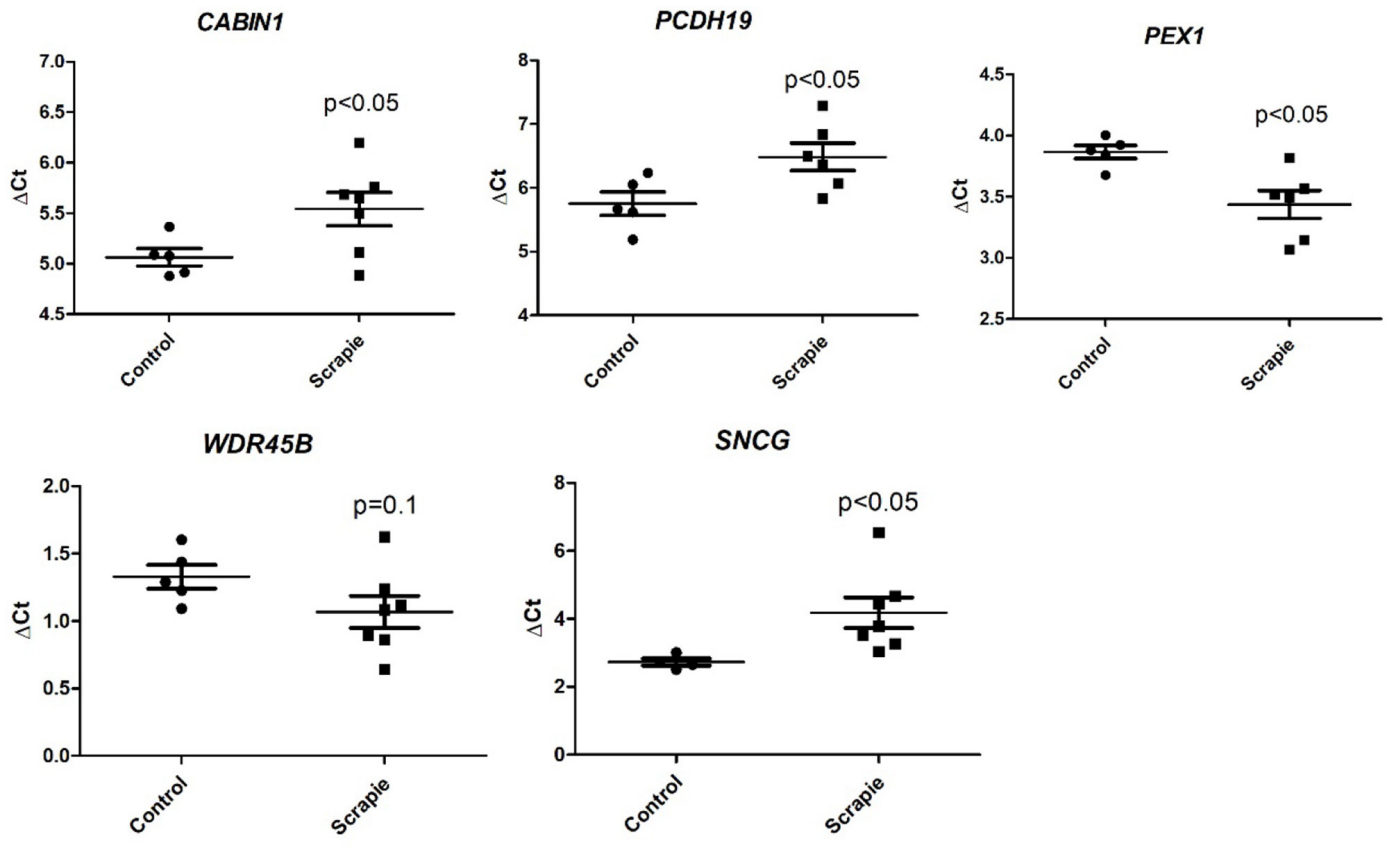
Gene expression profile of significant differentially expressed genes (*CABIN1, PCDH19, PEX1, SNCG*, and *WDR45B*). Data are shown in terms of relative gene expression (ΔCt) between control and scrapie animals.

As shown in Tables 2, 3 and Figure 8, significant changes were found between the control and scrapie animals in the expression of four genes (*PCDH19, SNCG, PEX1*, and *CABIN1*) and a trend toward significance in the expression of gene *WDR45B*.

### Correlation Between Differentially Methylated Gene Expression, PrP^Sc^ Accumulation, and Prion-Related Lesions

**Figure 9:**
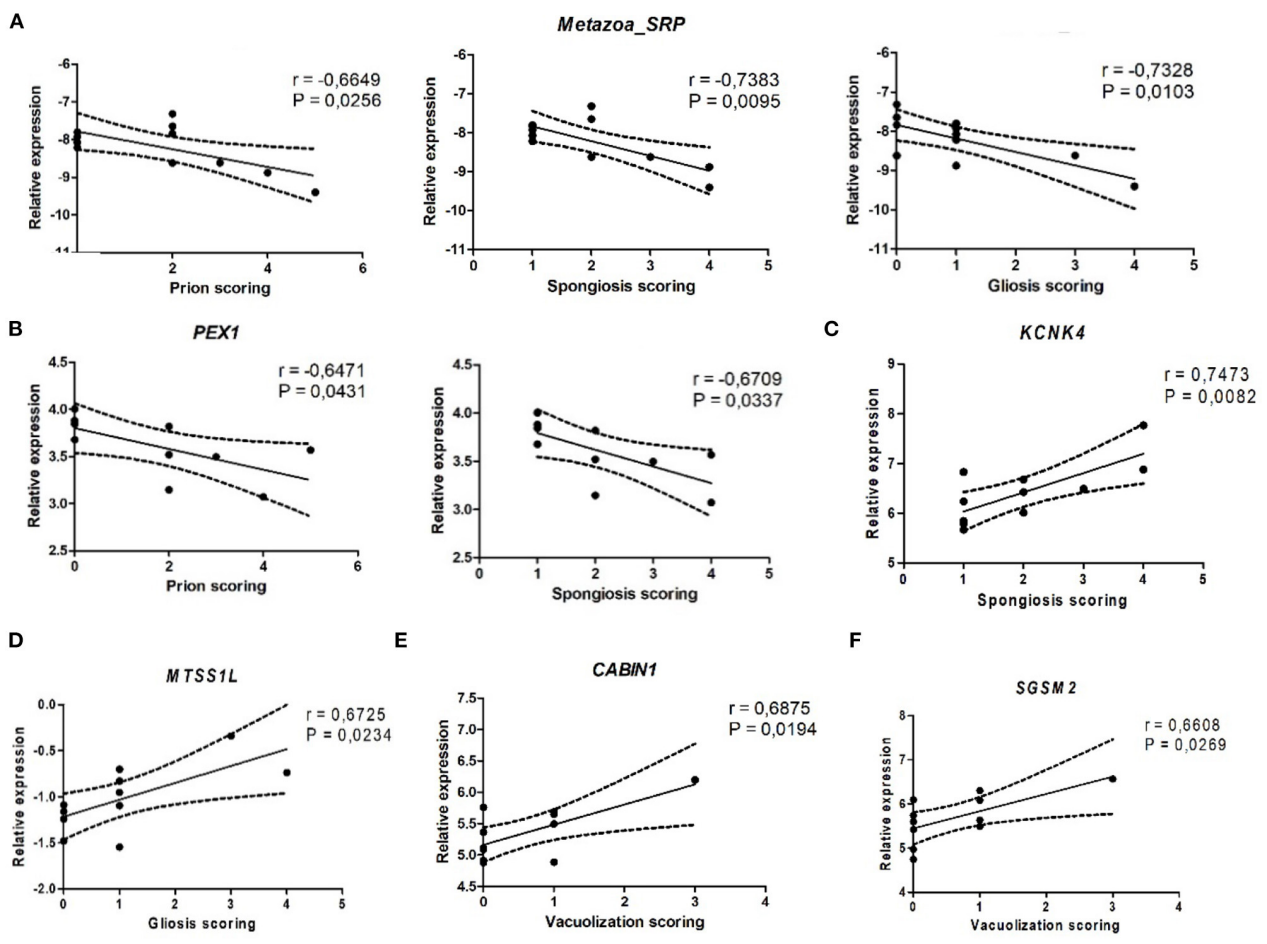
Correlation between PrP^Sc^ deposition, spongiosis, gliosis, and vacuolization scores and the expression of *Metazoa_SRP* **(A)**, *PEX1* **(B)**, *KCNK4* **(C)**, *MTSS1L* **(D)**, *CABIN1* **(E)**, and *SGSM2* **(F)**.

In order to find a possible association between DMG expression, PrP^Sc^ deposits, and prion-related lesions (spongiosis, gliosis, and vacuolization) (24), a correlation analysis was performed. A significant negative correlation was found between PrP^Sc^ accumulation and the expression of *PEX1* (*r* = −0.6471, *p* = 0.0431) (Figure 9B) and *Metazoa_SRP* (*r* = −0.6649, *p* = 0.0256) (Figure 9A). Regarding spongiosis, a significant negative correlation with *PEX1* (*r* = −0.6709, *p* = 0.0337) (Figure 9B) and *Metazoa_SRP* (*r* = −0.7383, *p* = 0.0095) (Figure 9A) expressions and a positive correlation with *KCNK4* expression (*r* = 0.7473, *p* = 0.0082) (Figure 9C) were also observed. *Metazoa_SRP* expression was as well negatively correlated with gliosis (*r* = −0.7328, *p* = 0.0103) (Figure 9A), and this prion-related lesion also showed a significant positive correlation with *MTSS1L* expression (*r* = 0.6725, *p* = 0.0234) (Figure 9D). Finally, a positive correlation between vacuolization and the expression of *CABIN1* (*r* = 0.6875, *p* = 0.0194) (Figure 9E) and *SGSM2* (*r* = 0.6608, *p* = 0.0269) (Figure 9F) was obtained.

## Discussion

DNA methylation has been studied as a possible epigenetic regulatory mechanism in the pathogenesis of several neurodegenerative diseases. DNA methylation might have a role in the progression and pathways linked to AD (38) and PD (39). These diseases are also identified as prion-like diseases because they share common pathogenic mechanisms with prion diseases, such as the accumulation of misfolded proteins in the CNS (40). These facts suggest that DNA methylation may also have a role in prion diseases.

To the best of our knowledge, and in contrast to AD (38), PD (41), and ALS (42), only one study has analyzed the genome-wide methylation profile in prion diseases, and this was performed in peripheral blood of sCJD patients (23). We report here the first WGBS study carried out in the CNS of any prion disease model. The study was performed in ovine classical scrapie, a natural animal model of prion disease. After neuroinvasion, PrP^Sc^ deposits in this form of scrapie are first observed in the spinal cord and obex, and from there they spread to the cerebellum, diencephalon, and prefrontal cortex (43). A previous work from our group revealed the similar intensity of PrP^Sc^ immunohistochemical signals in the obex (tissue selected for classical scrapie diagnosis), cervical spinal cord, and thalamus, although there was some variability between individuals showing a lesser degree of injury in some cases (44). In the present study, we have analyzed thalamus-derived DNA from a set of individuals whose obex was used in a previous transcriptomic analysis (5) and compared our methylation results with the reported expression changes. Although these techniques have been performed in different areas of the CNS, we believe that this approach would still be appropriate because previous validation of the expression changes observed in the array by qPCR showed similar results in the obex and diencephalon (thalamus and hypothalamus) (37). The sample size used in this study is limited (four scrapie vs. four control tissues) but adequate for the WGBS approach (45–49).

Although no significant differences were seen in global methylation levels between scrapie and control animals, significant correlations of methylation levels with PrP^Sc^ accumulation and prion-related lesions were evidenced in the scrapie group. However, significant correlations were seen in the total percentage of methylated cytosine and in motifs other than CpG, which was the most frequent methylated motif. Furthermore, this study allowed the identification of a great number of DMRs between the control and scrapie animals.

GO enrichment and KEGG pathway analyses of DMGs revealed an enrichment of several molecular and cellular functions in scrapie-affected animals. The most prominent enriched functions include intracellular signal transduction, transmembrane transport, protein and cellular binding, calcium signaling pathway, cAMP signaling pathway, cholinergic synapse, circadian entrainment, and apoptosis pathway. Interestingly, the PrP^C^ seems to have a role in all these processes. Several hypotheses suggest that PrP^C^ modulates components involved in proliferation, cell adhesion, transmembrane signaling, differentiation, and trafficking signaling pathways (50). In addition, PrP^C^ might also regulate synaptic transmission and plasticity, preserving normal synaptic structure and function (51). Regarding circadian entrainment, the role of PrP^C^ in sleep homeostasis and sleep continuity has been described (51). Stimulating the cAMP signaling pathway, PrP^C^ seems to promote cell survival, neurite outgrowth (52), and myelin maintenance (51). It has also been reported that PrP^C^ may regulate intracellular calcium homeostasis and exert control on mitochondria-associated apoptotic signaling (52). Therefore, the enrichment observed in all these functions when the PrP^C^ has lost its function due to its conversion to the pathological prion protein suggests a possible epigenetic regulation of all these processes.

Among the epigenetic mechanisms, DNA methylation has been described to participate in gene expression regulation. It is known that the position of the methylation in the transcriptional unit influences its relationship to the control of gene expression (53). Depending on whether the methylation is in a promoter, exon, or intron region, the effect on gene expression is different. Methylation in the promoter region is commonly associated with gene repression, whereas methylation in the gene body (exons) is associated with gene activation (53). Also, there is an inverse correlation between DNA methylation of the first intron and gene expression that could be due to the presence of intronic enhancers interacting with the promoters (54). With the purpose of studying the effect of DNA methylation on gene expression in Scrapie, we selected, among all the identified significant DMRs, a series of genes that have important functions in the nervous system and in some neurodegenerative diseases. Of all the selected genes, significant changes between the control and scrapie animals were found in the expression of five genes: *PCDH19, SNCG, WDR45B, PEX1*, and *CABIN1*.

The expression of the gene encoding Protocadherin 19 (*PCDH19*) was downregulated. This gene is located on chromosome X and belongs to the protocadherin family involved in signal transduction at synapses and in the establishment of neuronal connections. Protocadherins are mainly expressed in the CNS and participate in neuronal development, migration, segregation, and synaptic plasticity. Accordingly, the highest expression levels of *PCDH19* are found in the nervous system, although it is also expressed in several embryonic and adult tissues such as the kidney, lungs, and trachea (55). This gene has a role in the proliferation of neuronal progenitors, the formation of neuronal circuits, and the regulation of neuronal activity (56). Moreover, *PCDH19* seems to participate in GABAergic transmission, migration, and morphological maturation of neurons (57). Autism, intellectual disabilities, and epilepsy are related to defects in the expression or function of protocadherins, and mutations in *PCDH19* gene cause early infantile epileptic encephalopathy-9 (EIEE9) in humans (55). To the best of our knowledge, *PCDH19* methylation has only been studied in hepatocellular carcinoma in which hypermethylation of the promoter region correlated with a downregulation of the gene expression was observed (58). Here, a decrease in the expression of this gene was in accordance with promoter hypermethylation in naturally infected scrapie animals. Given the important role of *PCDH19* in the maintenance of neuronal homeostasis and neuronal connections, its downregulation observed in scrapie animals could be associated with earlier onset and/or the augmented progression of the prion disease.

Another DMG was γ-synuclein (*SNCG*), a member of the synuclein family that encompasses an important class of intrinsically disordered neural proteins. It is known that this protein has pathogenic implications in both neurodegeneration and cancer (59). γ-Synuclein is physiologically expressed by astrocytes in the human nervous system stimulating the cell cycle and participating in the expression and release of extracellular brain-derived neurotrophic factor (BNDF) (60). γ-Synuclein may also inhibit the aggregation propensity of α-synuclein, a protein present in Lewy bodies whose aggregation is a hallmark in PD (61). In contrast, overexpression of γ-synuclein in the neurons of transgenic mice induces a severe neurodegenerative pathology characterized by substantial depletion of neurofilaments in neuronal processes and, ultimately, death of motor neurons (62). In AD patients, γ-synuclein is also increased extracellularly in the brain and cerebrospinal fluid (60). In addition, upregulation of γ-synuclein is considered a prognostic marker in neurodegenerative conditions (62) and multiple invasive cancers (63, 64). Taking all these data into consideration, the downregulation and promoter hypermethylation observed in naturally infected scrapie animals suggest a possible neuroprotective role of this gene at astrocytes that are known to be involved in prion replication and spread.

WD repeat domain 45B (*WDR45B/WIPI3*) belongs to the WIPI protein family and was overexpressed in scrapie animals. All human WIPI proteins (WIPI1, WIPI2, WIPI3, and WIPI4) are known to have a role in the control of the autophagy process. In fact, *WDR45B* seems to participate in the formation of functional autophagosomes (65). Autophagy is a quality control mechanism for the degradation of misfolded proteins and damaged organelles and plays an important role in the maintenance of neural homeostasis (66). Dysregulation of the autophagy process has been described in different natural and experimental animal models of prion diseases (67–70) and other neurodegenerative diseases (71). In addition, *WDR45B* is associated in humans with a neurodevelopmental syndrome characterized by spastic quadriplegia, epilepsy, intellectual disability, and cerebral hypoplasia (72). *WDR45B* knockout mice display deficits and cognitive defects (66). The fact that neuronal homeostasis and autophagy are dysregulated in scrapie could possibly explain the *WDR45B* upregulation observed in naturally infected scrapie animals. The increased expression of this gene could be a host response that aims for the proper maintenance of the autophagic capacity and balanced neuronal homeostasis to slow down the prion disease progression.

Peroxisomal biogenesis factor 1 (*PEX1*) is a gene encoding the peroxin 1 protein that is involved in the peroxisome biogenesis specifically in importing peroxisomal matrix proteins (73) and was upregulated in scrapie animals. Peroxisomes are important metabolic organelles that contribute to cellular lipid metabolism and redox balance (74). Even though peroxisomes are present in most mammalian cell types, their contribution to the CNS function is related to the biosynthesis of ether phospholipids. These are important components of myelin in the synthesis of docosahexaenoic acid (DHA), which plays an important role in nervous system signaling, and to the degradation of toxic compounds and D-amino acids, which would protect brain structures and modulate synaptic signaling, respectively (75). Neurological diseases such as AD, autism, ALS (75), and PD (74) present dysfunction of peroxisomes or dysregulation of peroxisomal metabolites. As an example, patients with pronounced AD pathology display an inefficient peroxisome transport between neurites and soma and dysregulation in peroxisomal lipid metabolism. These alterations contribute to AD pathology aggravating disease progression. However, whether they are a secondary phenomenon or play a causative role in AD pathogenesis remains to be determined (74). In our study, we detected an upregulation of *PEX1* gene in naturally infected scrapie animals that were significantly correlated with spongiosis and PrP^Sc^ accumulation, suggesting that peroxisome activity might as well be compromised in scrapie. Although further research is needed, this finding could also indicate a possible role of peroxisomes in the pathogenesis of prion diseases.

Calcineurin-binding protein 1 (*CABIN1*) is a gene that acts as a calcium-dependent repressor of calcineurin in the CNS (76). Calcineurin is a serine/threonine phosphatase widely expressed in different cell types and structures including neurons, where it is involved in synaptic transmission and neurotransmitter release (77). The chronic aberrant activation of this protein in neurons contributes to synaptic dysfunction in AD. In contrast, calcineurin inhibition can improve synaptic morphology in AD mouse models (78). Synaptic dysfunction and synaptic loss are as well prominent and early events in prion diseases (51). Moreover, calcineurin activation mediated by human prion protein triggers neuronal cell death (79). Altogether, these data suggest that the downregulation of *CABIN1* observed in scrapie animals, which showed a significant association with vacuolization, could trigger calcineurin activation contributing, along with other pathogenic mechanisms, to the synaptic impairment and neuronal cell death observed in scrapie disease.

Although we found no significant changes in the expression of *Metazoa_SRP* (Metazoan signal recognition particle RNA) between the control and scrapie animals, we observed a significant negative correlation between the expression of this gene and PrP^Sc^ accumulation, spongiosis, and gliosis. *Metazoa_SRP* is a noncoding RNA (ncRNA). This type of molecule is abundantly expressed in the brain, and some of them have been reported to be dysregulated in neurodegenerative diseases. In addition, ncRNAs have been proposed as potential biomarkers for neural disorders (80, 81). Deregulation of different types of ncRNAs, namely, microRNAs, long noncoding RNAs, and circular RNAs, has been described in AD (82, 83) and PD (84, 85) and also in prion diseases including scrapie (86, 87). Although further research is warranted, the association of *Metazoa_SRP* with prion-related lesions could indicate a possible implication of this ncRNA in scrapie pathology.

Finally, in addition to the expression study of genes with functions in the nervous system and in some neurodegenerative diseases, we compared genes previously described to be differentially expressed in scrapie (5) with our set of identified DMRs finding that some of these genes also harbored DMRs. This fact suggests that DNA methylation could also be implicated in the expression of these previously described genes.

In conclusion, our study shows a potential regulatory role of DNA methylation in prion pathology. We identified many DMRs between the control and scrapie animals, some of them belonging to genes with possible neuroprotective roles against neurodegeneration (*SNCG* and *WDR45B*) and to genes that may facilitate or contribute to scrapie disease progression (*PCDH19, PEX1*, and *CABIN1*). Additionally, an enrichment in a variety of molecular and cellular functions in which the PrP^C^ is involved was found in naturally infected scrapie animals, supporting the idea that epigenetic regulation could have an important role in prion diseases. Due to the limitations of sample size and regions studied, replication of the study using a larger number of animals and other CNS areas is warranted in the future.

## Data Availability Statement

The raw and processed sequencing data generated for this study have been deposited in NCBI's Gene Expression Omnibus (88) and are accessible through GEO Series accession number GSE184767 (https://www.ncbi.nlm.nih.gov/geo/query/acc.cgi?acc=GSE184767).

## Ethics Statement

The animal study was reviewed and approved by Comisión Ética Asesora para la Experimentación Animal de la Universidad de Zaragoza.

## Author Contributions

AH: validation, formal analysis, investigation, and writing—original draft. AS and SS: validation, formal analysis, and investigation. BR: software, formal analysis, and investigation. OL-P: investigation. PZ and JB: funding acquisition. HF: formal analysis and investigation. RB: supervision, project administration, and funding acquisition. JT: conceptualization, methodology, investigation, project administration, and funding acquisition. IM-B: conceptualization, methodology, investigation, writing—review and editing, supervision, project administration, and funding acquisition. All authors reviewed the final version of the manuscript.

## Funding

AH and OL-P were supported by research grants from Gobierno de Aragón (Order IIU/2023/2017 and C012/2014) co-financed by the European Social Fund. This research was partially funded by the following projects: AGL2015-67945-P funded by MINECO; RTI2018-098711-B-I00 funded by MCIN/AEI/10.13039/501100011033 FEDER Una manera de hacer Europa; reference group A19-20R funded by the Government of Aragón co-financed with FEDER 2014-2020 Construyendo Europa desde Aragón and the European Regional Development Fund (ERDF), and EFA 148/16 REDPRION funded by Spain-France-Andorra Cooperation Program (POCTEFA). POCTEFA aims to reinforce the economic and social integration of the French–Spanish–Andorran border. Its support is focused on developing economic, social, and environmental cross-border activities through joint strategies, favoring sustainable territorial development.

## Conflict of Interest

The authors declare that the research was conducted in the absence of any commercial or financial relationships that could be construed as a potential conflict of interest.

## Publisher's Note

All claims expressed in this article are solely those of the authors and do not necessarily represent those of their affiliated organizations, or those of the publisher, the editors and the reviewers. Any product that may be evaluated in this article, or claim that may be made by its manufacturer, is not guaranteed or endorsed by the publisher.
