# Supplementary material for: Genome-Wide Methylation Profiling in the Thalamus of Scrapie Sheep
Source: Front Vet Sci. 2022 Feb 16;9:824677. doi: 10.3389/fvets.2022.824677 (PMC8888973; doi:10.3389/fvets.2022.824677)
Supplement: Supplementary file 1 [file Data_Sheet_1.docx]

Supplementary Material

**Supplementary Table S1.** Age and clinical status of the sheep used in the WGBS study. The number of days the animal showed scrapie symptoms from clinical diagnosis to sacrifice is shown in brackets. Scoring of prion deposition, spongiosis and gliosis. The extent of scrapie lesions was evaluated semi-quantitatively and scored on a scale ranging from 0 to 5 (0 = absence of lesions or immunolabelling, 5 = substantial lesions or immunolabelling throughout the region).

| **Animal** | **Age (years)** | **Clinical status** | **PrP^Sc^** | **Spongiosis** | **Gliosis** |
| --- | --- | --- | --- | --- | --- |
| C1 (O-1076) | 4 | control | 0 | 0 | 1 |
| C2 (O-1075) | 5 | control | 0 | 0 | 0.75 |
| C3 (O-1086) | 5 | control | 0 | 0 | 1 |
| C4 (O-1078) | 5 | control | 0 | 0 | 0.75 |
| Sc1 (O-859) | 5 | Clinical (35 days) | 0.5 | 1.75 | 3 |
| Sc2 (O-941) | 4 | Clinical (85 days) | 1.75 | 2.75 | 1.75 |
| Sc3 (O-996) | 5 | Clinical (unknown) | 1.25 | 2.25 | 3 |
| Sc4 (O-758) | 6 | Clinical (70 days) | 1.5 | 3 | 2.75 |

**Supplementary Table S2.** Specific primers used in the qPCR study (Fw= Forward primer; Rv= Reverse primer).

| Gene | Primer sequence |
| --- | --- |
| *A1BG* | Fw: 5’ –AGCAAATCCATTCAGCGCAG- 3’  Rv: 5’ –AATCGTCGTTGCCCTTGTAGC- 3’ |
| *CABIN1* | Fw: 5’ –AGGCTCCAACCATGATTCGA- 3’  Rv: 5’ –AATCGGTCGTGCTTCTGCA- 3’ |
| *CAPN1* | Fw: 5’ –CAGGATCATCAGCAAACACAAAGA- 3’  Rv: 5’ –ATCCGGTTCCATAGGATGTTGAA- 3’ |
| *CD81* | Fw: 5’ –TCCATGAGACGCTTAACTGCTG- 3’  Rv: 5’ –CAAATTACTGATGACGTTGCCG- 3’ |
| *GSTA4* | Fw: 5’ –AAGGAGAGAACCCTGATTGACATGT- 3’  Rv: 5’ –GGAAAGGATGCATGATAAGCAGTT- 3’ |
| *INTS1* | Fw: 5’ –ATCCGTGGTCCCGATTGAAG- 3’  Rv: 5’ –TCTTGGCCAGGTACATGAGG- 3’ |
| *KCNK4* | Fw: 5’ –CGAACCCTGACACCAACTCAA- 3’  Rv: 5’ –GTTGCCATAGCCGATGGTTGT- 3’ |
| *MAD1L1* | Fw: 5’ –TGGCAGAGCTGAAGAAGCAGGT- 3’  Rv: 5’ –TACATGGACGTCAGCCGGTACT- 3’ |
| *Metazoa_SRP* | Fw: 5’ –TCAATATGGTGACCTCCCGG- 3’  Rv: 5’ –TCTCGCTATGTTGCCCAGG- 3’ |
| *MTSS1L* | Fw: 5’ –GGCACGAGATCAAGAAGAAGT- 3’  Rv: 5’ –GCCACGCTCCTCAGTCAG- 3’ |
| *NARS* | Fw: 5’ –ATCTTGCTGTGTCGCTTTCCC- 3’  Rv: 5’ –CCCACGATCTCACCAACATTG- 3’ |
| *PCDH19* | Fw: 5’ –GAGTGACCAGACCGACAGTGAA- 3’  Rv: 5’ –AGGCATCTGAGATCCCATGGA- 3’ |
| *PEX1* | Fw: 5’ –TGAGTACCAATCTGCTGCAGAA- 3’  Rv: 5’ – GGCTATGAGTGATGTGCTCCA- 3’ |
| *PLA2G5* | Fw: 5’ –TATGGCTTCTACGGCTGTCA- 3’  Rv: 5’ –TGCGACCCTGTATCTGTAGG- 3’ |
| *PLCL2* | Fw: 5’ –CAGTACCCCACCATGGAGTT- 3’  Rv: 5’ –CATACACGGCATCTGCATTC- 3’ |
| *PSMG4* | Fw: 5’ –CTTAGCCAGGAAGACCAGCAAG- 3’  Rv: 5’ –AGAACTTCTCCGGAAAGGCCT- 3’ |
| *SNCG* | Fw: 5’ –TTTCATCGGCGTCAATAAGA- 3’  Rv: 5’ –TTGGCAATAGAGAAGCCCTT- 3’ |
| *SNX33* | Fw: 5’ –TGGTTGGTGAAATGTTCGCC- 3’  Rv: 5’ –GCCTTTCTGCAGGTGGATGAT- 3’ |
| *WDR45B* | Fw: 5’ –TCCAAGTTTCAGGTCCCCTC- 3’  Rv: 5’ –TGTCATCGGTCATCTCCAGG- 3’ |
| *WSCD2* | Fw: 5’ –CGTGGACAAGTGTGTGGACT- 3’  Rv: 5’ –CAGAGCTGCTCGTCCTCTCT- 3’ |

**Supplementary Table S3.** Raw data and quality summary.

| Sample | Raw Reads | Total Rawdata (Gb) | Effective Rate (%) | Clean Bases ^1^ (Gb) | Clean Q20 ^2^ (%) | Clean Q30 ^3^ (%) | Clean GC ^4^ (%) | BS Conversion Rate ^5^ (%) |
| --- | --- | --- | --- | --- | --- | --- | --- | --- |
| C1 | 1,152,563,414 | 172,9 | 95,38 ± 0,77 | 18,34 ± 5,45 | 97,61 ± 0,38 | 94,02 ± 0,68 | 20,27 ± 0,08 | 99,88 ± 0,009 |
| C2 | 1,072,851,262 | 160,9 | 95,09±0,83 | 17,02 ± 5,06 | 97,51 ± 0,39 | 93,77 ± 0,72 | 20,10 ± 0,07 | 99,88 ± 0,010 |
| C3 | 873,628,710 | 131 | 95,70 ± 0,82 | 13,94 ± 4,05 | 97,72 ± 0,38 | 94,19 ± 0,72 | 21,07 ± 0,08 | 99,89 ± 0,007 |
| C4 | 1,052,915,966 | 157,9 | 95,62 ± 0,74 | 16,80 ± 4,93 | 97,71 ± 0,36 | 94,23 ± 0,65 | 20,24 ± 0,07 | 99,88 ± 0,007 |
| Sc1 | 832,778,776 | 124,9 | 95,90 ± 0,74 | 13,31 ± 3,86 | 97,76 ± 0,37 | 94,30 ± 0,70 | 21,07 ± 0,08 | 99,89 ± 0,009 |
| Sc2 | 902,552,816 | 135,4 | 95,66 ± 0,83 | 14,41 ± 4,22 | 97,72 ± 0,37 | 94,20 ± 0,71 | 20,69 ± 0,07 | 99,88 ± 0,007 |
| Sc3 | 835,274,724 | 125,3 | 95,26 ± 1,42 | 11,96 ± 3,79 | 97,63 ± 0,27 | 93,87 ± 0,84 | 20,84 ± 0,10 | 99,89 ± 0,015 |
| Sc4 | 845,463,068 | 126,8 | 95,06 ± 1,47 | 12,10 ± 4,07 | 97,56 ± 0,27 | 93,73 ± 0,85 | 20,75 ± 0,09 | 99,89 ± 0,009 |

^1^ Proportion of clean bases in raw bases

^2^ Proportion of bases with quality score higher than 20

^3^ Proportion of bases with quality score higher than 30

^4^ Proportion of G and C in all the bases

^5^ Bisulfite conversion rate for C converting to T

**Supplementary Table S4.** Coverage depth of cytosine sites.

| Sample ^1^ | C_covg Mean ^2^ | C(Mb) ^3^ | CG(Mb) ^4^ | CHG(Mb) ^5^ | CHH(Mb) ^6^ | Mean C ^7^ | Mean CG ^8^ | Mean CHG ^9^ | Mean CHH ^10^ |
| --- | --- | --- | --- | --- | --- | --- | --- | --- | --- |
| C1 | 11.6 | 12,269 | 474 | 2659.1 | 9,135.8 | 3.46 | 74.25 | 0.56 | 0.63 |
| C2 | 10 | 10,630.8 | 413.4 | 2299.2 | 7,918.2 | 3.38 | 73.83 | 0.48 | 0.54 |
| C3 | 10.4 | 10,978.3 | 440.2 | 2415.1 | 8,123.0 | 3.43 | 74.27 | 0.43 | 0.48 |
| C4 | 10.3 | 10,869.4 | 425.4 | 2368.6 | 8,075.3 | 3.42 | 74.08 | 0.49 | 0.56 |
| Sc1 | 10.5 | 11,134.9 | 442.1 | 2443.5 | 8,249.3 | 3.83 | 75.23 | 0.79 | 0.90 |
| Sc2 | 10.3 | 10,893.3 | 431.5 | 2392.2 | 8,069.6 | 3.41 | 73.37 | 0.48 | 0.53 |
| Sc3 | 10.6 | 11,205.6 | 438 | 2434.8 | 8,332.8 | 3.65 | 75.79 | 0.65 | 0.74 |
| Sc4 | 9.1 | 9,596.7 | 390.4 | 2113.7 | 7,092.5 | 3.41 | 71.63 | 0.47 | 0.53 |

^1^ Sample: sample name

^2^ C_covgMean: The mean coverage of all the cytosine sites

^3^ C(Mb): The base number mapped onto the genome cytosine sites

^4^ CG(Mb): The base number mapped onto the genome CG-context cytosine sites

^5^ CHG(Mb): The base number mapped onto the genome CHG-context cytosine sites

^6^ CHH(Mb): The base number mapped onto the genome CHH-context cytosine sites

^7^ MeanC: The average methylation level of all the genome cytosine sites

^8^ MeanCG: The average methylation level of all the genome CG-context cytosine sites

^9^ MeanCHG: The average methylation level of all the genome CHG-context cytosine sites

^10^ MeanCHH: The average methylation level of all the genome CHH-context cytosine sites

**Supplementary Table S5.** Genomic methylation profile.

| Samples | mC ^1^ (%) | mCpG ^2^ (%) | mCHG ^3^ (%) | mCHH ^4^ (%) |
| --- | --- | --- | --- | --- |
| C1 | 4.71 | 41.07 | 2.1 | 3.02 |
| C2 | 4.53 | 40.76 | 2.13 | 2.78 |
| C3 | 4.42 | 41.58 | 2.16 | 2.56 |
| C4 | 4.76 | 41.43 | 2.37 | 2.98 |
| Sc1 | 6.71 | 43.24 | 4.27 | 4.95 |
| Sc2 | 4.36 | 39.95 | 1.99 | 2.65 |
| Sc3 | 5.91 | 43.08 | 3.38 | 4.14 |
| Sc4 | 4.54 | 40.85 | 2.44 | 2.7 |
| Control | 4.60 ± 0.16 | 41.21 ± 0.37 | 2.19 ± 0.12 | 2.84 ± 0.21 |
| Scrapie | 5.38 ± 1.12 | 41.78 ± 1.64 | 3.02 ± 1.01 | 3.61 ± 1.13 |
| All | 4.99 ± 0.85 | 41.50 ±1.14 | 2.61 ± 0.80 | 3.22 ± 0.86 |

^1^ Percent of methylated cytosines in all genomic cytosines

^2^ Percent of methylated cytosines in CG regions

^3^ Percent of methylated cytosines in CHG regions

^4^ Percent of methylated cytosines in CHH regions


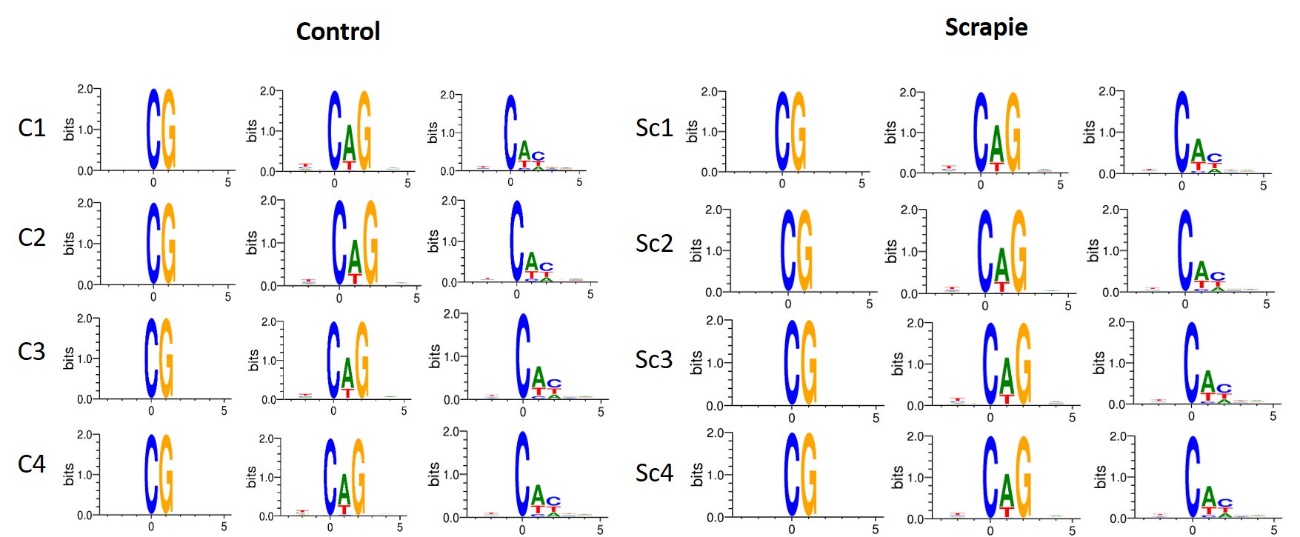


**Supplementary Figure S1.** Sequence preferences flanking the 9 pb sequences around methylated C sites in CG, CHG and CHH contexts in control (C1, C2, C3, C4) and scrapie (Sc1, Sc2, Sc3, Sc4) animals. The horizontal axis represents the base position, whereas the vertical axis shows the entropy of the base.


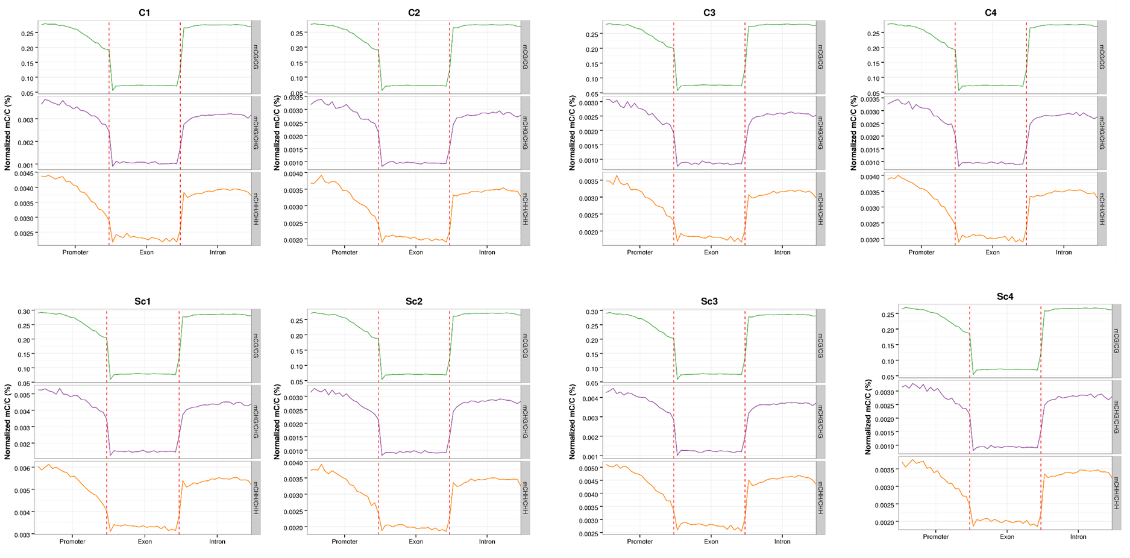


**Supplementary Figure S2.** Distribution of methylation level in various genomic features (Promoter, Exon and Intron) in control (C) and Scrapie (Sc) samples.
